# Editorial: Current insights in cancer metabolism and T cell based tumor immunity

**DOI:** 10.3389/fimmu.2025.1566491

**Published:** 2025-02-18

**Authors:** Takashi MaruYama, Takashi Tanikawa, Jian Gu

**Affiliations:** ^1^ Mucosal Immunology Unit, National Institute of Health, National Institute of Dental and Craniofacial Research (NIDCR), Bethesda, MD, United States; ^2^ Faculty of Pharmacy and Pharmaceutical Sciences, Josai University, Sakado, Japan; ^3^ Hepatobiliary Center, The First Affiliated Hospital of Nanjing Medical University and Research Unit of Liver Transplantation and Transplant Immunology, Chinese Academy of Medical Sciences, Nanjing, China

**Keywords:** cancer metabolism, cancer immunology, immune checkpoint inhibitor, tumor biology, glycolysis

Cancer metabolism is a key factor in elucidating the communication between tumor cells and immune cells. For example, during tumor cell proliferation, increased glycolysis results in the production of large amounts of L-lactate and TGF-beta by tumor cells, which can hinder tumor immunity by promoting the generation of regulatory T cells (Tregs) within the tumor microenvironment (1). Treatment with 2-Deoxyglucose, an inhibitor of glycolysis, has been shown to enhance anti-tumor immunity by reducing Treg generation.

Notably, anti-PD-1 therapy has been shown to activate T cells while also promoting glycolysis in the tumor microenvironment. In some patients, anti-PD-1 antibody treatment can lead to an increase in the proliferation of Tregs in the tumor microenvironment, thereby limiting the efficacy of immune checkpoint inhibitors. Xuekai et al. have suggested that a combination of anti-PD-1 and anti-TGF-beta therapies may offer a new solution to overcome resistance to anti-PD-1 therapy. Wang et al. have further elucidated how TGF-beta in the tumor microenvironment contributes to resistance against anti-PD-1 therapy. For instance, PD-1 plays a critical role in immune evasion by esophageal cancer cells, which express high levels of TGF-beta. TGF-beta produced by esophageal cancer cells induces an M2-phenotype in tumor-associated macrophages, reducing the population of CD8+ T cells involved in specific anti-tumor responses through the PD-1/PD-L1 pathway. Additionally, TGF-beta indirectly promotes immune suppression by activating Tregs in the tumor microenvironment.

Moreover, lipid peroxidation significantly influences the regulation of the tumor microenvironment (Xiao et al.). A lipid-enriched tumor microenvironment can induce an M2-type phenotype in tumor-associated macrophages through the upregulation of CD36, a fatty acid transporter. Tregs within the tumor microenvironment also express CD36, making them well adapted to the lipid-enriched environment. Shen et al. identified differences in immune activity, lipid biosynthesis, and drug metabolism between low- and high-risk survival groups of estrogen receptor-positive (ER+BR) breast cancer patients. The analysis revealed that high-risk patients express high levels of ALOX15, a gene associated with lipid metabolism, which is positively correlated with tumor size and vascular invasion. The authors have suggested that modulation of lipid metabolism may enhance the efficacy of anticancer therapies.

In conclusion, metabolism within the tumor microenvironment plays a crucial role in the regulation of both tumor growth and immune response. Lipid metabolism exerts a significant influence on the characteristics of the tumor microenvironment and the functionality of immune cells. A comprehensive understanding of these metabolic interactions could facilitate the development of innovative therapeutic strategies that combine metabolic modulation with immune checkpoint inhibitors to enhance anti-tumor immunity.
